# *C1orf127* deficiency causes cardiac abnormalities by disrupting the regulation of transcription initiation and interactions with RNA-binding proteins

**DOI:** 10.1016/j.gendis.2026.102105

**Published:** 2026-02-23

**Authors:** Yan Chen, Cancan Yao, Kun Yu, Bangguo Wu, Yuan Zhang, Xiangyu Zhou

**Affiliations:** a Obstetrics and Gynecology Hospital of Fudan University, Fudan University Shanghai Medical College, Shanghai 200090, China; b The Fourth Affiliated Hospital of Soochow University, Suzhou Dushu Lake Hospital, Soochow, Jiangsu 215123, China; c NHC Key Lab of Reproduction Regulation, School of Pharmacy, Fudan University, Shanghai 200237, China; d Shanghai First Maternity and Infant Hospital, Tongji University School of Medicine, Shanghai 201204, China

Left-right (LR) asymmetry is crucial for proper embryonic development in vertebrates. Disrupted LR organization results in laterality disorders, particularly heterotaxy and situs inversus totalis, conditions strongly associated with congenital heart disease (CHD),^1^ Unlike simple CHD, laterality defects coupled with CHD often follow a monogenic inheritance pattern and result in severe phenotypes (complex CHD) that cannot be effectively corrected surgically. Cilia-driven fluid flow during embryogenesis is crucial for establishing LR asymmetry in mammals, as evidenced by the approximately 50% incidence of laterality defects in individuals with primary ciliary dyskinesia. The gene *C1orf127* (orthologous to murine *Gm572*) encodes a secretory protein. Sequence prediction and comparison revealed that this protein possesses a 22-amino acid N-terminal signal peptide and a substantial disordered region. Prior to our study, this represented the entirety of our knowledge regarding the protein's function. Recent studies by Dong et al and Szenker-Ravi et al reported a genome-wide significant enrichment of recessive loss-of-function mutations (p.E69X, p.R113X, p.R193X and p.Q404RfsTer84) in *C1orf127* among CHD patients,^2^^,^^3^ However, these studies did not include further functional investigations. While the genetic evidence from CHD cohorts is substantial, the functional role of C1orf127 during embryonic heart development remains largely unexplored.

Although loss-of-function mutations in C1orf127 have been reported in CHD patients, neither knockdown of *c1orf127* in zebrafish nor in Xenopus displayed defective LR phenotypes,^3^ which presents a significant challenge to the notion of C1orf127 as a CHD-causing gene. Against this backdrop, and to precisely elucidate the role of *Gm572* in heart development, we generated a novel knockout (KO) mouse model using CRISPR/Cas9 gene editing (Fig. S1A). Sanger sequencing of the mutant allele confirmed a five-base deletion in exon 2 (c.109_113delGCGCA, NM_001368835) (Fig. S1B and Table S1), leading to a frameshift and protein premature translation termination (p.A37WfsTer20) (Fig. S1C). At postnatal day 7, the proportion of homozygous (hom) pups deviated significantly from the expected Mendelian ratio (Fig. S1D). By embryonic (E) day 17, more than 30% of hom mutants exhibited embryo resorption and developmental malformations (Fig. 1A). Histological staining on the remaining, outwardly normal-appearing embryos revealed that 33.75% of *Gm572* ^KO/KO^ embryos developed CHD, compared to *Gm572* ^WT/KO^ and WT (Fig. 1B). Notably, approximately 18.1% of *Gm572* ^KO/KO^ embryos displayed abnormal cardiac LR patterning (Fig. 1B). A small proportion of *Gm572* ^KO/KO^ mice survived to adulthood without obvious abnormalities. Echocardiographic analysis of these healthy survivors showed no significant changes in key cardiac parameters, such as left ventricular ejection fraction and fractional shortening (Fig. S2). *Gm572* ^KO/KO^ male mice were fertile and capable of producing offspring with *Gm572* ^KO/KO^ female mice. Collectively, these findings suggest that *Gm572* deficiency causes cardiac LR defects in mice embryos, which provide a strong evidence to support a causal link between *C1orf127* mutations and human CHD.

**Figure 1:**
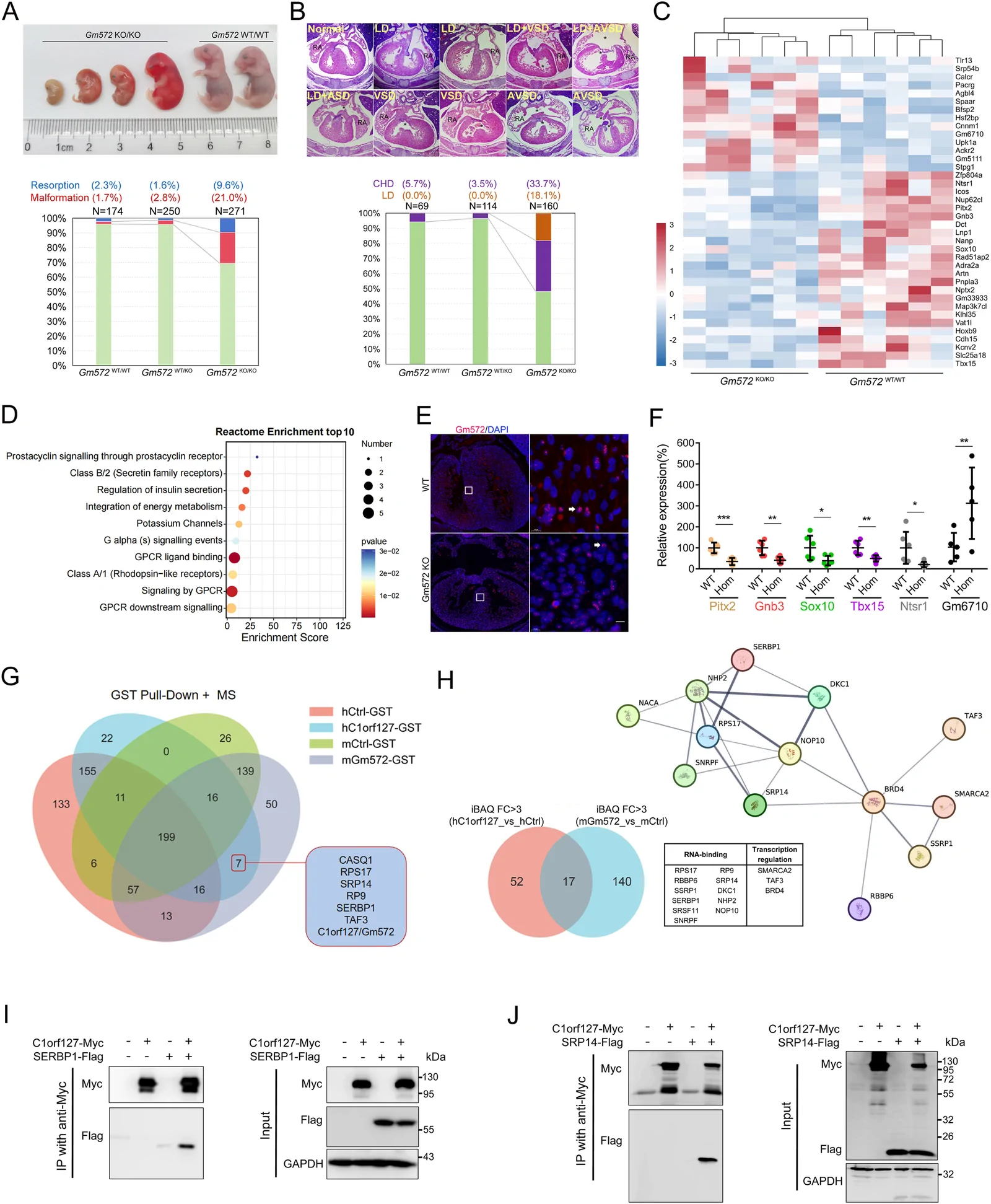
*Gm572 knockout* causes cardiac abnormalities by disrupting the interactions with RNA-binding proteins and regulation of transcription initiation. **(A)** Representative images (up) and quantification (bottom) of embryo resorption and malformations observed in *Gm572*^KO/KO^ mice at E17. **(B)** Representative images (up) and quantification (bottom) of laterality defects (LD) and congenital heart disease (CHD) observed in *Gm572*^KO/KO^ mice at E17. VSD, ventricular septal defect; ASD, atrial septal defect; AVSD, atrioventricular septal defect (simultaneous occurrence of ASD and VSD). **(C)** Heatmap showing differential gene expression profiles in E12.5 *Gm572*^KO/KO^ mice compared to WT, as determined by RNA-sequencing (*n* = 6). **(D)** Enrichment analysis of Reactome pathways for 38 differentially expressed genes (DEGs) (Fisher's exact test; *P* < 0.05). **(E)***Gm5*72 mRNA was visualized by fluorescence *in situ* hybridization at E17. Scar bar, 10 μm. **(F)** The dys-regulation of six candidate genes from RNA-seq analysis, including *Pitx2, Gnb3, Sox10, Tbx15, Ntsr1 and Gm6710*, was further validated using real-time quantitative PCR. ∗∗∗*P* < 0.001, ∗∗*P* < 0.01, ∗*P* < 0.05 (two-tailed Student's *t* test; *n* = 5). **(G)** Interactome mapping of human (h) C1orf127 and murine (m) Gm572 in the heart using GST pull-down coupled with mass spectrometry (MS). Seven overlapping proteins specifically interact with both hC1ORF127 and mGm572 compared to the corresponding Ctrl-GST. **(H)** Protein–protein interaction network of 17 overlapping candidate proteins (iBAQ FC > 3) visualized using the STRING database and networkD3 R package. Eleven RNA-binding proteins and three transcription regulator proteins were displayed. **(I, J)** Representative images of co-immunoprecipitation validation for SERBP1 (I) and SRP14 (J) *in vitro*. Anti-Myc immunoprecipitation assays were performed in HEK293T cells followed by immunoblotting with the indicated antibodies. Representative images from three independent experiments were displayed.

To investigate the effect of *Gm572* deficiency on the cardiac transcriptome, we performed RNA-seq analysis on heart tissue from *Gm572* ^KO/KO^ and WT mice (*n* = 6) at E12.5 (Fig. S3A and S3B). Compared to WT littermates, *Gm572* ^KO/KO^ mice exhibited 38 differentially expressed genes (Fold-change > 2, *P* < 0.05) (Fig. 1C). Notably, multiple genes encoding DNA-binding transcription factors including *Pitx*2 (paired-like homeodomain transcription factor 2), *Tbx15* (T-box transcription factor 15) and *Sox10* (SRY-box transcription factor 10) were significantly downregulated, while *Gm6710* (orthologous to human zinc finger protein 17, ZNF17) was upregulated (Fig. 1C). Several G-protein coupled receptor (GPCR) signaling related protein encoding genes, including *Ackr2*, *Adra2a*, *Calcr*, *Gnb*3, and *Ntsr1,* were dysregulated in *Gm572* ^KO/KO^ mice. Reactome analysis revealed significant enrichment in pathways related to “GPCR ligand binding”, “Regulation of insulin secretion”, “Potassium Channels” and “Calcitonin-like ligand receptors” (Fig. 1D). These findings suggest that *Gm572* deficiency potentially disrupts transcription initiation and GPCR signaling pathway during heart development. The mRNA level of *Gm572* was not affected in *Gm572* ^KO/KO^ mice as assessed by RNA-sequencing and quantitative PCR (qPCR) (Fig. S3C). Due to the lack of an antibody suitable for detecting the endogenous protein, we performed fluorescence *in situ* hybridization and found that *Gm572* signals were uniformly distributed in the atrium and ventricle at E14.5 (Fig. 1E). Real-time qPCR then confirmed the dysregulation of five key genes: *Pitx2*, *Tbx15*, *Sox1*0, *Gnb*3 and *Gm6710* (Fig. 1F). Previous studies have established that Nodal signaling induces asymmetric Pitx2 expression on the left side, a key determinant of left-right asymmetry, thereby governing embryonic laterality.^4^ We further validated the reduced *Pitx2* expression in the hearts of *Gm572* ^KO/KO^ mice by qPCR at E9.5 day (Fig. S3D), a critical window for cardiac tube looping and chamber formation. These results indicate that *Gm572* deficiency disrupts left-right patterning by impairing Pitx2 expression.

To define the interaction landscape of human (h) C1orf127 and murine (m) Gm572, we conducted GST pull-down assays coupled with mass spectrometry (MS) analysis. GST-tagged hC1orf127 (aa 22–399) and mGm572 (aa 28–399) were cloned into pGEX-6P-1 vectors (Fig. S4, S5), respectively, and recombinant proteins were purified and then incubated with embryonic heart tissue lysates (human: 14 gestational weeks; mouse: E12.5). All the procedures in the study were approved by the Ethics Committee of Obstetrics and Gynecology Hospital of Fudan University (kyy2022–126). Signed informed consent was obtained from the participants before study commencement. Compared with hCtrl-GST and mCtrl-GST, we identified 29 and 57 proteins that specifically bound to hC1orf127-GST and mGm572-GST (Fig. 1G), respectively, with seven proteins overlapping including CASQ1 (calsequestrin 1), RPS17 (ribosomal protein S17), SRP14 (signal recognition particle 14), SERBP1 (SERPINE1 mRNA binding protein 1), RP9 (RP9 pre-mRNA splicing factor) and TAF3 (TATA-box binding protein associated factor 3) (Fig. 1G). The identification of hC1ORF127/mGm572 as bait proteins strengthened the reliability of the data. Significantly, four of the overlapping proteins, RPS17, SERBP1, RP9 and SRP14, possess RNA-binding activity and are known to interact with each other. Furthermore, we profiled 69 and 157 candidate proteins (fold-change of intensity-based absolute quantification [iBAQ] values > 3) in hC1orf127 and mGm572-GST groups, respectively, relative to the corresponding Ctrl-GST controls (Fig. 1H). Gene Ontology (GO) analysis revealed that the 17 overlapping proteins were significantly enriched for RNA-binding proteins and ribonucleoprotein (RNP) complexes (Fig. S6A; Fig. 1H), implicating C1orf127 in the regulation of ribosome biogenesis in eukaryotes. Five overlapped proteins including SERBP1, SRP14, CASQ1, TAF3 and RPS17 were then selected for co-immunoprecipitation validation (Fig. 1I, J; Fig. S6B–S6D). C1orf127 appears to participate in other cellular physiological processes, including calcium ion storage and transcription initiation, through its interactions with CASQ1 and TAF3. Collectively, the interactome data suggest that C1orf127 interacts with multiple RNA-binding proteins and is involved in the regulation of transcription initiation.

Two recent studies intensively reported recessive *C1orf127* mutations in a total of 22 affected individuals with heterotaxy and congenital heart defects.^2^^,^^3^ We also analyzed the trio-based whole exome sequencing data of 236 Chinese patients with laterality defects and CHD. We identified biallelic *C1orf127* missense mutations in two unrelated affected individuals (Fig. S7A). In a sporadic heterotaxy (Htx) case, we detected compound heterozygous mutations in *C1orf127* (c.626A>T:p.Y209F, c.2138G>A:p.R713H) (parental samples were unavailable for segregation analysis) that met our filtering criteria (frequency < 0.01 in gnomAD v2.1, protein-altering variants, recessive model) (Fig. S7B). Minor allele frequencies (MAFs) were 0.000 for p.Y209F and 9.61e-5 for p.R713H according to GnomAD v4.1 database. In one situs inversus totalis (SIT) trio with complex CHD, affected individuals carried biallelic mutations in *C1orf127* (c.92C>T:p.T31M, c.1439C>T:p.A480V, NM_001170754), which were validated by Sanger sequencing (Fig. S7C). MAFs were 1.70e-4 for p.T31M and 6.33e-5 for p.A480V according to GnomAD v4.1 database. Since knockdown of *c1orf127* in zebrafish did not affect LR patterning, generating point-mutation knock-in (KI) models represents an efficient strategy to further investigate the pathogenicity of these missense mutations. Nonetheless, we provide additional genetic evidence supporting the role of C1orf127 in establishing cardiac asymmetry within the Chinese Han population.

Given that Gm572 is reported to be expressed in the LR organizer (LRO) of mice,^3^ we further examined the effect of Gm572 deficiency on motile cilia by establishing cultures of mouse nasal respiratory epithelial cells. However, we did not observe obvious defects in the 9 + 2 ultrastructure of cilia by transmission electron microscopy analysis (Fig. S8A), and cilia density and beating frequency was not significantly altered in Gm572^KO/KO^ mice. While the current evidence suggests that Gm572 deficiency may contribute to CHD via a non-ciliary mechanism, future investigations are needed to explore potential alterations in ciliary signaling pathways or sensory functions to support this hypothesis. Especially, the observed disruption of Pitx2 expression and GPCR signaling in *Gm572* ^KO/KO^ mouse hearts could potentially stem from aberrant ciliary-driven nodal flow. Moreover, *Gm572* was stably expressed in the mouse heart from embryonic stage to the adult period, with minimal expression observed in the kidney, liver, and lung (Fig. S8B). Through *in situ* hybridization assay, we have shown that Gm572 mRNA signals were uniformly distributed in the heart as aforementioned. These findings suggest that the biological function of Gm572 extends beyond the embryonic LRO and may also play an important role in the adult period.

Although the precise mechanisms underlying heart defects in C1orf127-deficient patients remain to be elucidated,^2^^,^^3^^,^^5^ we generate a novel mice model that supports the causality between *C1orf127* mutations and human complex CHD. Thus, preimplantation genetic testing (PGT) technology can be utilized to prevent the transmission of *C1orf127* mutations to offspring, offering effective precision medicine consultation for mutation carriers and for families planning subsequent pregnancies. We generate the first interaction network for C1orf127 in the heart, thereby offering new insights into its protein function. Our results indicate that C1orf127 deficiency leads to cardiac abnormalities potentially by disrupting the regulation of transcription initiation and interactions with RNA-binding proteins.

## CRediT authorship contribution statement

**Yan Chen:** Investigation, Data curation. **Cancan Yao:** Validation, Investigation. **Kun Yu:** Investigation. **Bangguo Wu:** Investigation. **Yuan Zhang:** Investigation, Data curation. **Xiangyu Zhou:** Writing – original draft, Supervision, Funding acquisition, Conceptualization.

## Ethics declaration

For studies of affected individuals and their families, written informed consent was obtained from all participants prior to the start of the study. All procedures in the study were approved by the Medical Ethics Committee of Obstetrics and Gynecology Hospital of Fudan University (kyy2024-154) (Shanghai, China). For animal studies, all experimental protocols were conducted in accordance with the institutional ethical guidelines and approved by Institutional Animal Care and Use Committee of Laboratory Animal Center Fudan University (2024-FCKYY-172).

## Funding

This work was supported by the National Natural Science Foundation of China (No. 82171845, 82371865), and grants from Natural Science Foundation of Shanghai (China) (24ZR1458300), and National Key Research and Development Project (China) (2018YFA0801300).

## Conflict of interests

The authors declare no competing interests.

## References

[1] Fakhro K.A., Choi M., Ware S.M. (2011). Rare copy number variations in congenital heart disease patients identify unique genes in left-right patterning. Proc Natl Acad Sci U S A.

[2] Dong W., Jin S.C., Sierant M.C. (2011). Recessive genetic contribution to congenital heart disease in 5,424 probands. Proc Natl Acad Sci U S A.

[3] Szenker-Ravi E., Ott T., Yusof A. (2015). CIROZ is dispensable in ancestral vertebrates but essential for left-right patterning in humans. Am J Hum Genet.

[4] Ryan A.K., Blumberg B., Rodriguez-Esteban C. (1998). Pitx2 determines left-right asymmetry of internal organs in vertebrates. Nature.

[5] Alsamri M.T., Alabdouli A., Iram D. (2021). A study on the genetics of primary ciliary dyskinesia. J Clin Med.

