# Supplementary material for: C1orf127 deficiency causes cardiac abnormalities by disrupting the regulation of transcription initiation and interactions with RNA-binding proteins
Source: Genes Dis. 2026 Feb 23;14(2):102105. doi: 10.1016/j.gendis.2026.102105 (PMC13634566; doi:10.1016/j.gendis.2026.102105)
Supplement: Supplementary file 1 [file mmc1.docx]

***C1orf127* deficiency causes cardiac abnormalities by disrupting the regulation of transcription initiation and interactions with RNA-binding proteins**

**Supplementary Figure legends.**

**Figure S1.** Generation and Genotypic Validation of the Gm572 Knockout (KO) Mouse Model. (A) Strategy of generation *Gm572* KO mouse model using a CRISPR/Cas9 gene editing system. Sequences of two sgRNA were displayed. (B) Sanger sequencing of gDNA identified a 5-bp deletion at exon 2 in *Gm572* KO mice. (C) Sanger sequencing of cDNA confirmed a 5-bp deletion in the mutant allele that would lead to premature translation termination. (D) Genotype distribution of *Gm572* mice (WT/WT, WT/KO, KO/KO) at postnatal day 7.

**Figure S2.** **Echocardiographic Assessment of Cardiac Function in Surviving Adult** Gm572 **KO Mice.** Echocardiographic analysis of *Gm572* ^KO/KO^ mice (n=10) and their WT littermates (n=11) at the age of 3 months. The core cardiac parameters including left ventricular ejection fraction (EF, %), fractional shortening (FS, %), left ventricle posterior wall (LVPW, mm) and left ventricle volume (ul) were displayed; s, systolic; d, diastolic; two-tailed Student’s t test. The data are presented as the mean ± SEM; each mouse was assayed in triplicate.

**Figure S3.** Transcriptomic Profiling of Gm572 KO Mice Hearts at E12.5 Days. (A) Overview of gene expression in each heart sample of *WT* (n=6) and *Gm572* KO (n=6) mice by RNA-seq analysis. (B) Score plots of principal component analysis (PCA) of *WT* and *Gm572* KO. (C) The mRNA level of *Gm572* was not significantly downregulated in *Gm572* KO mice according to RNA-seq analysis (two-tailed Student’s t test; n=6, fold-change=1.19). (D) The Pitx2 expression was significantly reduced in *Gm572* ^KO/KO^ mice hearts at E9.5 day by using real-time quantitative PCR (Ordinary one-way ANOVA; n=3, **, p<0.01).

**Figure S4**. Interactome of Human (h) C1orf127 in Heart Tissue Through GST-Pull Down Coupled with Mass Spectrometry (MS) Analysis. (A) GST-tagged hC1orf127 (aa 22-399) was cloned into pGEX-6P-1 vector. (B) GST-tagged hC1orf127 recombinant proteins were purified and validated by coomassie blue staining. (C-D) Purified recombinant proteins (GST-Ctrl and GST-hC1orf127) incubated with human embryonic heart tissue lysates to perform GST pull-down and then visualized by Western blot (anti-GST) (C) and Silver staining (D) before MS analysis.

**Figure S5**. Interactome of Murine(m) Gm572 in Heart Tissue Through GST-Pull Down Coupled with Mass Spectrometry (MS) Analysis. (A) GST-tagged mGm572 (aa 28-399) was cloned into pGEX-6P-1 vector. (B) GST-tagged mGm572 recombinant proteins were purified and validated by coomassie blue staining. (C-D) Purified recombinant proteins (GST-Ctrl and GST-mGm572) incubated with mouse embryonic heart tissue lysates to perform GST pull-down and then visualized by Western blot (anti-GST) (C) and Silver staining (D) before MS analysis.

**Figure S6.** RNA-Binding Protein Enrichment and Co-IP Validated Protein Interactions for C1orf127/Gm572. (A) Gene Ontology (GO) analysis revealed that the 17 overlapping proteins were significantly enriched for RNA-binding proteins. 11 proteins including DKC1, NHP2, NOP10, RBBP6, RPS17, SNRPF, SRP14, SRSF11, SSRP1, RP9 and SERBP1 were classified into RNA-binding term. (B-D) Protein interaction of CASQ1 (B), RPS17 (C), and TAF3 (D) with C1orf127/Gm572 was further confirmed by Co-IP assay.

**Figure S7**. Identification of *C1orf127* Mutations in Two Patients with Laterality Defects and CHD. (A) Pedigrees of two families, illustrating the affected individuals and the segregation of *C1orf127* recessive variants in Family 2. (B-C) Sanger sequencing confirmed the identified missense mutations in *C1orf127* from whole exome sequencing (WES).

**Figure S8**. Ciliary Axoneme Ultrastructure and *Gm572* Expression Pattern in *Gm572* KO Mice. (A) Representative transmission electron microscopy (TEM) images of cross-sections of ciliary axonemes from *Gm572* ^KO/KO^ mice. Scale bar, 2 µm (Left Up) and 200 nm (Right Up). (B) RNA-seq analysis demonstrated that *Gm572* was stably expressed in the mouse heart from embryonic (E12.5 and 18 day) to the adult period (P16 week), with minimal expression observed in the kidney, liver, and lung.

**Supplementary Tables**

**Table S1.** Sequences of primers used for real-time qPCR assay and Sanger sequencing.

| Primers for genotyping (Gm572 KO model) |  |
| --- | --- |
| Gm572 GenoF | 5' CCATGCCCTTCGCTCTTC 3' |
| Gm572 GenoR | 5' GATGCCTTCTGCCTTCCA 3' |
| Primers for Sanger sequencing | |
| C1orf127-1439CF | 5' TGAAGAAGGGGCTTGGACC 3' |
| C1orf127-1439CR | 5' AGGGGACTTCCTGGAGACT 3' |
| C1orf127-2138GF | 5' GTTTCTCACCGTTCTCCCG 3' |
| C1orf127-2138GR | 5' GGCTGGGTAGCAACACTCC 3' |
| C1orf127-92CF | 5' CGTCTGGCTTGCCTGTGTT 3' |
| C1orf127-92CR | 5' CACACGACACACGCTCCCT 3' |
| C1orf127-626AF | 5' TCGTTCTTTCAGTTGCGTT 3' |
| C1orf127-626AR | 5' TCTTCTAGGTGCTATGCCC 3' |
| Primers for cDNA amplification | |
| Gm572-cDNA-F | 5' TGCTGTGTTCCCATGGATC 3' |
| Gm572-cDNA-R | 5' CGCCCCTTTTTGAAACATT 3’ |
| Pitx2-qPCR-F | 5' CGCCGTGTGGACCAACCTT 3’ |
| Pitx2-qPCR-R | 5' GGCCCAAAGCCATTCTTGC 3’ |

**Fig.S1**

**Fig.S2**

**Fig.S3**

**Fig.S4**

**Fig.S5**

**Fig.S6**

**Fig.S7**

**Fig.S8**
