# Supplementary material for: C1orf127 deficiency causes cardiac abnormalities by disrupting the regulation of transcription initiation and interactions with RNA-binding proteins
Source: Genes Dis. 2026 Feb 23;14(2):102105. doi: 10.1016/j.gendis.2026.102105 (PMC13634566; doi:10.1016/j.gendis.2026.102105)
Supplement: Supplementary file 2 [file mmc2.docx]

# Supplementary Methods

**Generation of Gm572 knockout (KO) mouse model.**

C57BL/6n female mice (7–8 weeks old) were used as embryo donors. Briefly, C57BL/6n female mice were superovulated by intraperitoneally injecting with PMSG and hCG, and then mated to C57BL/6n male mice. Fertilized embryos (zygotes) were collected from the oviducts, and Cas9 mRNA (100 ng/μL) plus sgRNA (50 ng/μL) targeting Gm572 were mixed and injected into the cytoplasm of the fertilized eggs with both pronuclei visible in CZB (Chatot–Ziomek–Bavister) medium. The injected zygotes were then cultured in Quinn’s Advantage cleavage medium (In-Vitro Fertilization, Inc.) containing SCR7 (50 µm, TOCRIS) for about 24 h, and 18–20 2-cell stage embryos were transferred into the oviduct of a pseudo-pregnant ICR female mouse at 0.5 dpc. All mice had access to food and water. All experiments were performed in accordance with Health Guide for the Care and Use of Laboratory Animals. Genomic DNA was extracted from tail tips, and Sanger sequencing-based genotyping was performed. Sequences of sgRNA and primers are provided in Supplementary data.

**Echocardiography**

Transthoracic echocardiography was performed in Gm572 KO and WT mice using a Vevo2100 system (FUJIFILM VisualSonics Inc.) with an MS400 transducer following anaesthesia with 2% isoflurane gas and 0.8 L/min oxygen. Echocardiography was used to assess left ventricle (LV) systolic and diastolic functions with a 2-dimensional guided M-mode ultrasound system (Acuson Sequoia, Siemens Medical Solutions, Mountain View, CA, USA). Two-dimensional images from a short-axis view of the LV were obtained at the level of the papillary muscles using a 15 MHz linear transducer at a paper speed of 100 mm/s. Echocardiography was performed before opening the chest (baseline reading) and during the course of the study for all groups. Three consecutive cardiac cycles were analysed, and the average values were used for data analysis.

**RNA sequencing**

Transcriptome libraries were prepared using a TruSeq RNA Sample Preparation kit (Illumina) and then sequenced using the Illumina HiSeq PE platform at 2 × 151-bp read lengths. The expression level of each transcript was calculated according to fragments per kilobase of exon per million mapped reads. Differentially expressed genes (DEGs) between groups were selected based on a log fold-change >2 and a false discovery rate FDR < 0.05. Gene ontology (GO) functional-enrichment and Kyoto Encyclopedia of Genes and Genomes pathway analyses were performed using GOATOLLS and KOBAS. DEGs were considered significantly enriched when the p value was less than 0.05 following Bonferroni’s correction

**TEM analysis**

Nasal tissues of Gm572 KO mice were collected and maintained in L-15 medium for video microscopy using a Leica inverted microscope (Leica DMI300B). Transmission electron microscopy (TEM) analysis was performed at the Department of Electron Microscopy, Fudan University. Briefly, the samples were fixed with glutaraldehyde (2.5% w/v) in 0.1 M sodium cacodylate buffer. Following fixation in osmium tetroxide (1% w/v), the samples were dehydrated through a graded ethanol series and embedded in epoxy resin. Sections were cut at 50–70 nm thickness and stained with 2% methanolic uranyl acetate and Reynold’s lead citrate for TEM (JEM-1400; Jeol, Tokyo, Japan).

**GST Pull down coupled with LC-MS/MS**

In this study, GST-tagged hC1orf127 (aa 22-399) and mGm572 (aa 28-399) were cloned into pGEX-6P-1 vectors. Inoculate 5ml of LB broth with ampicillin in a culture vial with a single colony of BL21 strain of E.coli. Cell lysate from BL21 expressing GST (Empty pGEX-6P-1) is used as control to compare with the cell lysate expressing GST fusion protein. Both samples are processed simultaneously. Prepare 50% slurry of Glutathione Sepharose 4B from 75% slurry and incubate the clarified crude cell lysate expressing GST or GST fusion protein with 50% slurry for 2-4 hours with gentle shaking. Wash the column three times with ice cold PBS. Add the clarified heart tissue lysate to the Glutathione Sepharose 4B column with immobilized GST/ GST fusion protein and incubate for 2-4 hours at 4ºC. Wash the column three times with ice cold PBS. Elute the interacting protein complex by incubating with 1glutathione elution buffer for 15 min at RT. The elutes thus obtained both from column containing both immobilized GST and GST fusion proteins are loaded adjacent to each other on a 10% SDS PAGE gel with appropriate molecular weight marker. Complexes eluted from the beads are resolved by SDS-PAGE and analyzed by silver/comassie staining. The protein bands excised from the gel is subjected to in-gel tryptic digestion protocol. The digested peptides mixture are analyzed by LC-MS/MS to identify the interacting proteins.

**RT-qPCR**

Total RNA was isolated from mouse heart tissues using the RNA Easy Fast Kit (DP451, Tiangen, Beijing, China) followed by first-strand cDNA synthesis. RT-qPCR was performed using the FastFire qPCR PreMix (SYBR Green) (FP207, TIANGEN) on an ABI StepOnePlus instrument. The delta-delta-Ct (ddCt) algorithm was utilized to analyze the relative change in gene expression by using the housekeeping gene GAPDH as the internal reference. The data are presented as the mean ± SEM of four independent experiments; each sample was assayed in triplicate in each experiment. RT-qPCR primer sequences are available in Supplementary data.

**Subjects**

Patients were diagnosed using X-ray, computed tomography (CT) and color ultrasonic scans at Obstetrics and Gynecology Hospital of Fudan University, Shanghai, China. For our studies on affected individuals and their families, signed and informed consent was obtained from all participants prior to the start of the blood drawing and heart tissue (aborted fetus 14 week) collection. All procedures in the study were approved by the Ethics Committee of Obstetrics and Gynecology Hospitall of Fudan University (KYY2024-154) (Shanghai, China).

**Statistical analysis**

Data are presented for individual samples as the mean ± SEM. Student’s two-tailed unpaired t-test or one-way ANOVA was used to determine the statistical significances of differences between the WT and mutant groups using GraphPad Prism software version 5.01 (GraphPad Software, Inc., La Jolla, CA, USA). Differences were considered statistically significant when p values were <0.05.
